# USP7 at PML Nuclear Bodies: A Protein Interaction Network Perspective

**DOI:** 10.3390/ijms27094106

**Published:** 2026-05-04

**Authors:** Sergey A. Silonov, Ekaterina S. Vedeshkina, Yakov I. Mokin, Dmitriy A. Sukailo, Eugene Y. Smirnov, Vladislav A. Reushev, Irina M. Kuznetsova, Konstantin K. Turoverov, Alexander V. Fonin

**Affiliations:** The Biomolecular Condensates and Membraneless Organelles Group, Institute of Cytology, Russian Academy of Sciences, 4 Tikhoretsky Ave., 194064 St. Petersburg, Russia; ekvedeshkina@yandex.ru (E.S.V.); mokinyakov@mail.ru (Y.I.M.); dima.sukailo@gmail.com (D.A.S.); e.smirnov@incras.ru (E.Y.S.); vareushev@gmail.com (V.A.R.); imk@incras.ru (I.M.K.); kkt@incras.ru (K.K.T.)

**Keywords:** USP7, PML nuclear bodies, LLPS, membraneless organelles, intrinsically disordered proteins, intrinsically disordered regions, protein–protein interactions

## Abstract

Ubiquitin-specific protease 7 (USP7/HAUSP) is one of the most studied deubiquitinating enzymes and plays a crucial role in regulating numerous cellular processes, making it a promising therapeutic target. In the nucleus, USP7 partially colocalizes with PML nuclear bodies (PML-NB)—multifunctional membraneless organelles involved in post-translational modifications and protein complexes assembly. The molecular basis and functional significance of this association remain uncharacterized. In this study, comparison of USP7 and PML interactomes revealed a significant overlap of 166 shared proteins. Functional enrichment analysis showed that USP7 and PML may operate within a common molecular context related to transcriptional regulation, chromatin remodeling, and DNA damage responses. Furthermore, these processes are also linked to cellular senescence and human aging (CellAge and GenAge databases). Focused analysis of overlaps between the USP7 interactome and core PML-NB proteins identified 61 proteins forming a dense “small-world” network. Most are prone to liquid–liquid phase separation, are intrinsically disordered, and serve as substrates for SUMOylation or ubiquitination. These findings not only expand our understanding of the molecular functions of USP7 but also highlight PML-NB as an important cellular context for investigating mechanisms associated with USP7 activity.

## 1. Introduction

Maintaining internal physiological balance (homeostasis) is a continuous and complex process in cell life. To prevent the loss of balance and subsequent detrimental intramolecular disruptions, cells strongly regulate homeostasis and use stress control mechanisms that detect disturbances and help resolve them [1]. The protein homeostasis (proteostasis) is achieved through the coordinated action of many proteins forming the proteostasis network, which, according to preliminary functional annotation data, consists of around 2000 proteins in human cells [2]. One of the proteostasis functional modules is the ‘ubiquitin-proteasome system’ (UPS) of protein degradation. Importantly, the UPS plays a crucial role in the degradation of over 80% of intracellular proteins [3,4,5]. A key mechanism of the UPS is the ubiquitination of proteins—a post-translational modification that regulates their stability, localization, and functional activity. This process is controlled by ubiquitinating or deubiquitinating enzymes (DUBs), which can selectively add or remove ubiquitin chains from protein substrates [6]. There are two main DUB classes: cysteine proteases and metalloproteases. The first class contains six families, the largest of which is the family of ubiquitin-specific proteases (USPs) [7].

The most studied member of the USP family is ubiquitin-specific protease 7 (USP7), also known as herpesvirus-associated ubiquitin-specific protease (HAUSP) [8]. USP7 plays a key role in the regulation of numerous fundamental cellular processes, including cell cycle control, DNA replication, mitosis, telomere maintenance, DNA damage response mechanisms such as nucleotide excision repair, double-strand break repair, and DNA damage bypass. Additionally, USP7 is involved in the regulation of apoptosis, epigenetic modifications, p53-dependent transcription, immune responses, and host–virus interactions [6,9]. USP7 is widely expressed in all tissue types of the human body. Dysregulation of USP7 is associated with numerous human diseases, including cancer, viral infections, and neurodegenerative disorders, highlighting its therapeutic potential in the treatment of various pathological conditions [10,11]. Among the pathological conditions associated with USP7 dysregulation, cancer has been most extensively studied. Increased USP7 expression has been observed in various types of malignancies, including lung cancer, osteosarcoma, squamous cell carcinoma of the oral cavity, melanoma, cervical cancer, nasopharyngeal cancer, liver cancer, glioblastoma, prostate cancer, breast cancer, and esophageal cancer [12].

**Figure 1:**
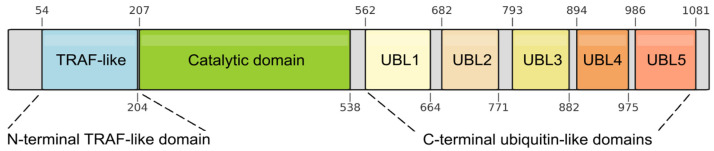
Schematic diagram of USP7 domains. The schematic shows the amino acid location of each domain. TRAF-like: tumor necrosis factor receptor-associated factor-like domain; catalytic domain; UBL1–5: C-terminal ubiquitin-like domains.

From its N- to C-terminus, USP7 contains a tumor necrosis factor receptor-associated factor-like (TRAF-like) domain, a catalytic domain, and five C-terminal ubiquitin-like (UBL1–5) domains (Figure 1). While the catalytic core remains conserved among USPs, each member achieves substrate specificity through distinct accessory substrate-binding domains [9].

Studies have shown that USP7 mediates the deubiquitination and stabilization of oncogenic proteins (MDM2, MDMX) and tumor suppressors (p53, PTEN, NF-κB, β-catenin, and FOXP3) [6]. Thus, overexpression or mutations in USP7 may be closely linked to the initiation and progression of tumorigenesis. Significant progress has been made to date in understanding the biological functions, structural characteristics, and drug development targeting USP7 [8].

USP7 is predominantly localized in the nucleus. Within the nucleus, the protein has both diffuse nucleoplasmic distribution and discrete nuclear foci, some of which colocalize with PML nuclear bodies (PML-NBs) [13]. PML-NBs are membraneless organelles that function as centers for post-translational modifications, protein complexes assembly, and sequestration of regulatory factors [14]. Their assembly is scaffolded by the promyelocytic leukemia (PML) protein, from which they take their name.

In addition to USP7 nuclear localization, a minor fraction of protein has also been detected in mitochondria and cytoplasm, showing its broad subcellular distribution [10]. The functional role of USP7 in nuclear foci and in PML bodies remains poorly understood.

It should be emphasized that full-length USP7 as well as its N-terminal (residues 1–208) and C-terminal (residues 560–1102) domains all form foci that are closely associated with PML-NBs [15]. However, the catalytic domain (residues 208–560) does not exhibit such an association, suggesting that USP7’s localization to PML-NBs is mediated by protein–protein interactions rather than the enzyme’s catalytic activity. It is proposed that USP7 primarily interacts with PML-I and PML-IV isoforms, causing a destabilizing effect on PML bodies [15]. DAXX and MDM2, which co-localize with PML in PML-NBs and physically interact with it, were also found to co-localize with USP7 upon endogenous expression of fluorescent fusion proteins [16,17,18].

USP7 has been identified as a “replisome-enriched SUMO deubiquitinase” essential for DNA replication [19]. Enriched at replication forks, USP7 deubiquitinates SUMOylated proteins and retains them at the DNA synthesis area. Notably, upon inhibition of USP7, SUMOylated proteins are displaced from replication factories, losing co-localization with PCNA foci, while their association with PML-NBs remains unaffected—further supporting the specificity of USP7’s function at replication forks [19]. It is noteworthy that PML-NBs can serve as hotspots for SUMOylation reactions in the cell nucleus [20], suggesting a functional link between these processes.

An important aspect of USP7 is its localization at the telomeric ends of chromosomes in both telomerase-positive and ALT-positive cell lines [21]. In telomerase-positive cells, USP7 plays a protective role by stabilizing the TPP1 protein. In ALT-positive cells, USP7 is associated with ALT-associated PML bodies (APBs) and promotes POT1 poly-ubiquitination and proteasomal degradation—a process that must be counteracted for proper ALT-mediated telomere maintenance [9].

It is known that USP7 interacts with the WDR79 protein, a key component of Cajal bodies [22]. This interaction leads to reduced ubiquitination of MDM2 and p53, thereby promoting their stabilization and extending their half-life. Such stabilization promotes cell proliferation, as both MDM2 and p53 are central regulators of the cell cycle and apoptosis [22]. Notably, Cajal bodies do not operate in isolation from PML-NBs: the two structures are often found in close nuclear proximity [23], an association partially mediated by interactions between coilin and PIASy [24]. They also share mobile protein components such as SMN [25] and are functionally coupled through the SUMOylation pathway—a mechanism critical for both PML-NB scaffold assembly and the Cajal body proteins regulation [25,26]. However, it is worth noting that to date, there was no information on the localization of USP7 specifically within Cajal bodies.

Brief summary information of USP7 subcellular localization and functional partnerships is presented in Table 1.

Growing evidence suggests that USP7 functions as a component of diverse molecular complexes, including PML-NBs. However, the functional significance of this association remains uncharacterized. This study analyzes a comparison of the interactomes of USP7 and PML, and provides a focused analysis of the overlaps between the USP7 interactome and core PML-NB-associated proteins, exploring their shared molecular functions, their connection to cellular and organismal aging, and the role of PML-NBs as a potential cellular context for understanding the mechanisms driven by USP7 activity.

## 2. Results and Discussion

### 2.1. Analysis of Proteins Common to the USP7 and PML Interactomes

**Figure 2:**
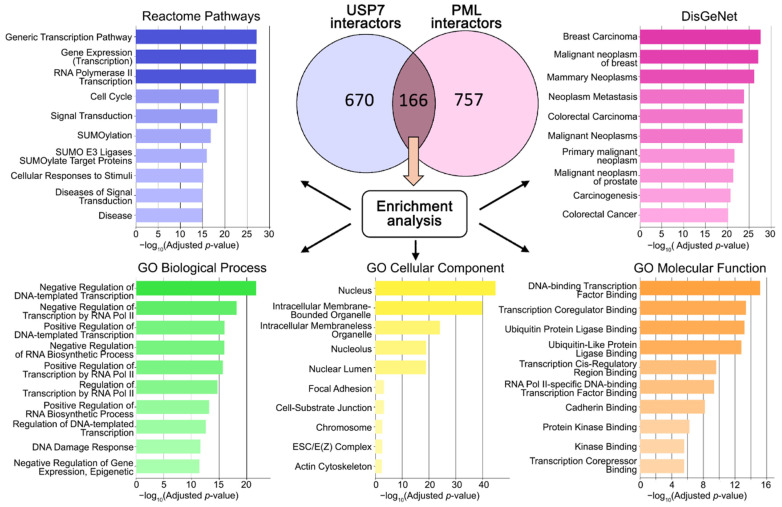
Enrichment analysis of the USP7 and PML interactomes overlap. Venn diagram illustrating the overlap between the USP7 interactome (836 proteins) and PML interactome (923 proteins) was constructed using data obtained from the BioGRID database. Enrichment analysis was performed on the 166 overlapping proteins. The following annotation databases were queried: Reactome Pathways, DisGeNET, and Gene Ontology (Biological Process, Cellular Component, Molecular Function). Results are displayed as horizontal bar charts representing the 10 terms with the lowest *p*-values. The complete results are provided in Supplementary Table S1.

USP7 is a deubiquitinase with a wide range of substrates and interaction partners. Accumulated data indicate that USP7 acts not only as an independent regulatory enzyme but also as a component of various molecular complexes. Given the close association between USP7 and PML nuclear bodies (PML-NBs), the first step in this study was to analyze the overlap between the USP7 and PML interactomes obtained from the BioGRID protein–protein interaction database (v 5.0). This analysis revealed a significant overlap of 166 shared proteins, which were further analyzed using enrichment analysis across various databases (Figure 2).

Functional enrichment analysis of proteins shared by the USP7 and PML interactomes revealed a significant predominance of biological pathways and gene ontology (GO) processes associated with transcriptional regulation (Figure 1). Enriched terms correspond primarily to both “positive and negative regulation of DNA-templated transcription”, particularly “RNA polymerase II-mediated transcription”, and “regulation of RNA biosynthetic processes”. In the GO molecular function category, enrichment was identified for several key groups: transcriptional regulation (binding to DNA, transcription factors, and coregulators), protein–protein interactions (ubiquitination, kinase binding), and cell adhesion (cadherins). This profile is characteristic of genes involved in epigenetic regulation and cellular architecture, suggesting the involvement of transcription factors or chromatin-remodeling proteins with additional cytoskeletal functions. Cellular component analysis (GO: cellular component) reveals predominantly nuclear localization of the shared proteins (nucleus, nucleolus, nuclear lumen), combined with chromatin-regulatory activity. Furthermore, enrichment of terms related to epigenetic regulation of gene expression and the DNA damage response suggests that these shared proteins may participate in chromatin-mediated transcriptional control and stress-sensitive regulatory pathways. Taken together, USP7 and PML may function within a common molecular context associated with transcriptional regulation, potentially involving chromatin-related mechanisms and transcriptional responses to DNA damage.

**Figure 3:**
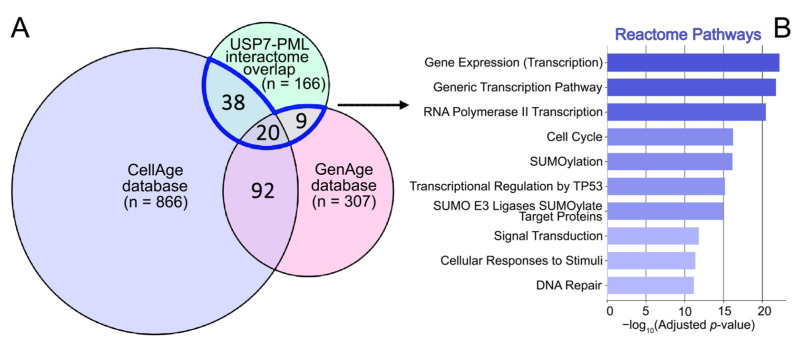
Involvement of USP7 and PML interactome overlap proteins in senescence and aging. (**A**) Venn diagram illustrates the overlap between the cellular senescence database (CellAge), the human aging database (GenAge), and the USP7 and PML interactome overlap proteins (166 proteins, see Figure 2). (**B**) Reactome Pathways enrichment analysis performed on proteins present in the intersection of all three datasets (CellAge, GenAge, USP7 and PML interactome overlap proteins). Results are displayed as horizontal bar charts representing the 10 terms with the lowest *p*-values. The complete results are provided in Supplementary Table S2.

A previous analysis by our group [33] of the PML protein role in natural cellular senescence and aging revealed a significant number of transcription factors among PML interactomes potentially involved in these processes. To test the association of shared USP7 and PML interactome proteins with cellular senescence and aging, a similar comparison analysis was performed with the CellAge and GenAge databases (Figure 3A).

We found that 67 of the 166 shared USP7 and PML interactome proteins were present in the CellAge or GenAge databases, suggesting a significant role for USP7 in its interaction with PML in cellular senescence and aging. A Reactome pathway enrichment analysis revealed that 39 of the 67 proteins overlapping with the CellAge and GenAge databases are involved in the regulation of gene expression, specifically transcription, suggesting a significant role for USP7 in transcription mediated by PML and PML-NBs (Figure 3B).

It is worth noting that the role of USP7 in transcription has already been identified, and several transcription factors are known USP7 substrates (p53, HMD2, Rb, FOX(O)3, FOX(O)4, PTEN, β-catenin, PPARγ, N-Myc) [34]. However, where exactly these processes occur within the cell remains unclear. Inhibition of USP7 activity is known to selectively kill senescent cells, partly by restoring p53 activity [35]. Conversely, USP7 inhibition can also induce senescence; genetic and pharmacological inhibition of USP7 induces senescence in melanoma patient-derived xenograft (PDX) models [36].

On the other hand, PML and PML-NBs play a significant role in senescence processes as structural and functional regulators. In primary fibroblasts obtained from both healthy donors and donors with premature aging syndromes (ataxia-telangiectasia and Cockayne syndrome), the size and number of PML-NBs increase upon transition to a senescent state, which may serve as a potential marker of cellular senescence [33]. Approximately 30–45% of the proteins interacting with PML and comprising PML-NBs are directly associated with senescence and aging processes, participating in the regulation of transcription factor activity, the response to DNA damage, and the suppression of apoptosis [33]. A recent study demonstrated that PML, through the assembly of the PML-mTOR-RONIN transcriptional complex, acts as an oncogene in triple-negative breast cancer (TNBC), where its silencing is sufficient to induce a beneficial senescence response [37]. Taken together, these findings and the existing literature suggest that USP7 in the context of PML-NBs may be associated with cellular senescence and organismal aging.

### 2.2. Colocalization of USP7 with PML Isoforms, UBC9, and MDM2

**Figure 4:**
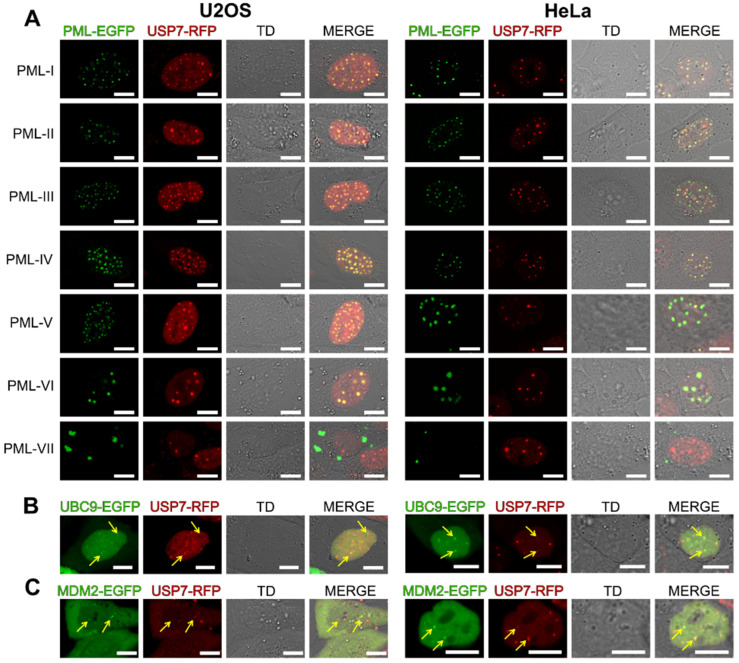
Colocalization of USP7 with PML isoforms, UBC9, and MDM2. (**A**) Localization of PML isoform-EGFP fusion proteins (PML-I–PML-VII) and USP7-RFP in U2OS and HeLa cells stably expressing USP7-RFP. (**B**,**C**) Localization of transiently expressed UBC9-EGFP (**B**) and MDM2-EGFP (**C**) relative to USP7-RFP nuclear foci. EGFP signal was detected at excitation 488 nm, emission 500–550 nm; RFP signal was detected at excitation 561 nm, emission 570–620 nm; TD: transmission detector. Yellow arrows indicate colocalization with USP7 foci. Scale bar: 10 μm.

PML protein is primarily localized to PML bodies, and USP7 forms discrete nuclear foci, a subset of which colocalizes with PML bodies. To study the spatial relationship between USP7 and the seven main isoforms of PML protein, we used chimeric fluorescent fusion constructs: USP7 tagged with red fluorescent protein (RFP) and each of the seven main PML isoforms (PML-I–PML-VII) fused to enhanced green fluorescent protein (EGFP). Stable HeLa and U2OS cell lines expressing USP7-RFP were created by lentiviral transduction, and individual PML-EGFP isoform constructs were introduced by transiently transfection via lipofection. Consistent with previous reports demonstrating that a fraction of USP7 associates with PML nuclear bodies (PML-NBs), colocalization of USP7 with PML-NBs was observed for isoforms PML-I through PML-VI. In contrast, PML-VII, which localizes exclusively to the cytoplasm and forms distinct cytoplasmic structures independent of nuclear body formation, did not colocalize with USP7 (Figure 4A). Given this finding, and considering that PML isoforms differ from one another only in their intrinsically disordered C-terminal regions, it is reasonable to propose that the previously reported interaction between USP7 and PML-IV [15] is mediated through the intrinsically disordered unique C-terminal to this isoform.

Given that recent work by Jung et al. [38] demonstrated that SLX4 stability within PML-NBs is maintained through USP7-mediated deubiquitination, and that SLX4 recruitment to PML-NBs depends on its SUMO-interacting motifs (SIMs) and their interaction with SUMOylated UBC9, we next examined whether UBC9 colocalizes with USP7 within nuclear foci. Using a fluorescent UBC9-EGFP construct introduced by transient transfection, we demonstrate that UBC9 colocalizes with USP7 nuclear foci (Figure 4B). This observation is consistent with the known role of USP7 as a SUMO deubiquitinase that counteracts ubiquitination of SUMOylated proteins at replication forks and within nuclear compartments [19], and further supports a functional link between the SUMO conjugation machinery and USP7 activity within PML-NBs.

Using the same experimental approach, we also demonstrate colocalization of MDM2 with USP7 nuclear foci (Figure 4C), in agreement with the well-established role of USP7 in stabilizing MDM2 through deubiquitination [13,15]. Of note, nuclear foci observed upon co-expression of USP7 with PML isoforms appeared markedly larger than those formed upon co-expression with UBC9 or MDM2. This morphological difference may reflect distinct modes of USP7 incorporation into PML-NBs depending on the interacting partner, and warrants further investigation.

### 2.3. PML-NB Hub Proteins Link USP7 to Nuclear Body Biogenesis

**Figure 5:**
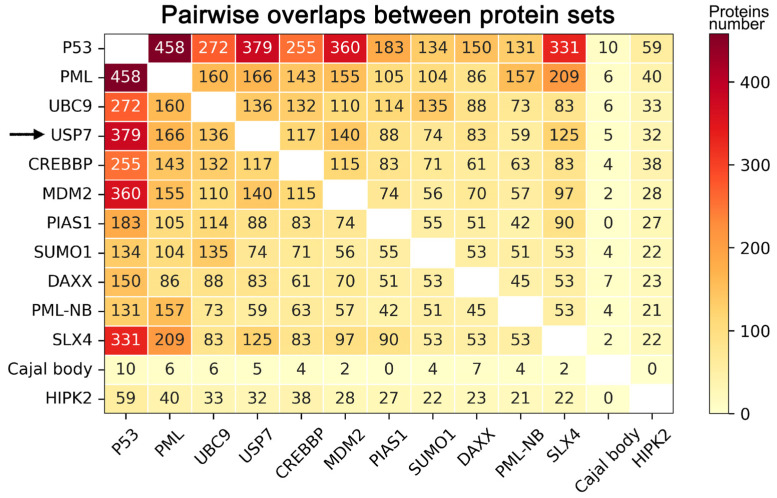
Pairwise overlaps between protein sets. Heatmap shows pairwise overlaps between the USP7 interactome, interactomes of core PML-NB-associated proteins (PML, UBC9, CREBBP, DAXX, SUMO1, MDM2, PIAS1, P53, HIPK2, SLX4), the PML-NB proteome (literature-derived from [39]), and the Cajal body proteome (UniProt KB, SL-0031). The black arrow points to the USP7 interactome row. All interactome data were obtained from the BioGRID database. Color intensity represents the number of shared proteins between each pair of sets.

Since PML protein is primarily localized to PML bodies, and USP7 forms discrete nuclear foci, a subset of which colocalizes with them [13], the next step of this work was to identify hub proteins potentially associated with this colocalization. To achieve this, we compared the interactomes of USP7 and core PML-NB-associated proteins (PML, UBC9, CREBBP, DAXX, SUMO1, MDM2, PIAS1, P53, HIPK2, SLX4) against the PML-NBs proteome (a literature-derived set of 205 proteins from [39]) and the Cajal body proteome (retrieved from UniProt KB; see Materials and Methods). This analysis revealed that USP7 is extensively interconnected with core PML-NB-associated proteins, sharing a substantial number of interaction partners with them (Figure 5).

Intersection of the resulting interactomes identified 61 proteins that interact with at least four core PML-NB-associated proteins with a BioGRID evidence score of ≥2. These proteins were subsequently subjected to a comprehensive bioinformatic characterization encompassing predictions of LLPS propensity, intrinsic disorder (IDP), SUMOylation, ubiquitination, disorder-promoting residues (DPR); results are summarized in Supplementary Table S3.

**Figure 6:**
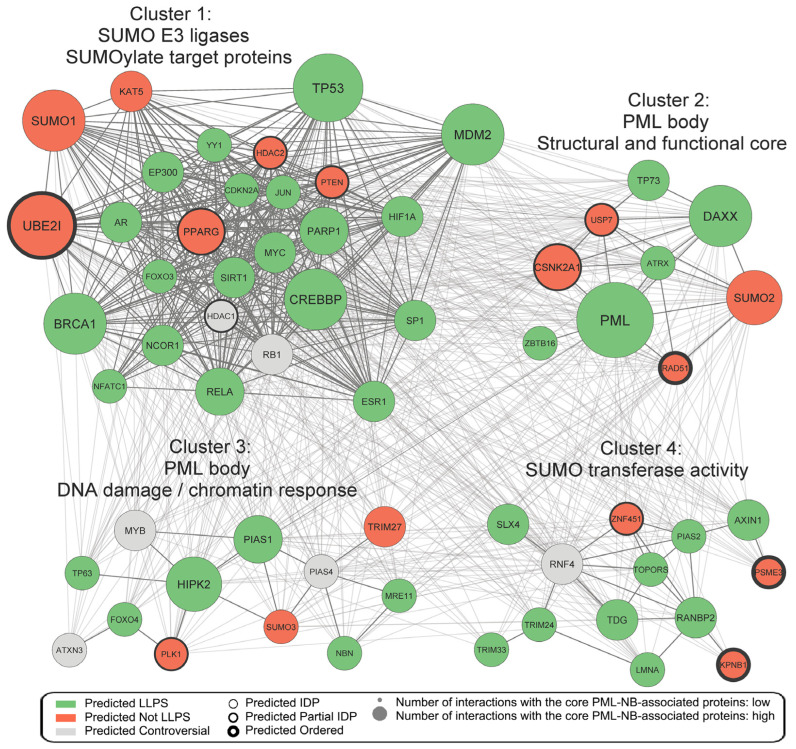
Network of identified hub proteins. The network displays clustering of 61 proteins identified in at least four interactomes of core PML-NB-associated proteins (PML, UBC9, CREBBP, DAXX, SUMO1, MDM2, PIAS1, P53, HIPK2, SLX4). Interaction data were retrieved from BioGRID (evidence score ≥ 2). The resulting interaction network was clustered using the MCODE algorithm in Cytoscape, revealing four major clusters. Cluster annotations were assigned based on functional enrichment analysis using the STRING database. Node size reflects the number of interactions with the core PML-NB-associated proteins. LLPS propensity was predicted using FuzDrop and PSPredictor. Node border width reflects the degree of intrinsic disorder as predicted by VSL2Score. The complete table are provided in Supplementary Table S3.

To visualize the degree of overlap among the identified hub proteins and to highlight their LLPS propensity and disorder content, a protein–protein interaction network was constructed (Supplementary Figure S1). The resulting network displays small-world topology with a high degree of interconnectivity between nodes, rendering direct interpretation challenging. Accordingly, the network was subjected to cluster analysis using the MCODE algorithm, which resolved four distinct clusters (Figure 6): (1) SUMO E3 ligases that SUMOylate target proteins; (2) PML body structural and functional core; (3) PML body chromatin organization and DNA damage response; (4) SUMO transferase activity. Notably, USP7 was assigned to cluster 2 (PML body structural and functional core), suggesting that USP7 could potentially play a significant role not only in the molecular processes occurring within PML-NBs, but also in the structural organization of PML bodies themselves.

**Figure 7:**
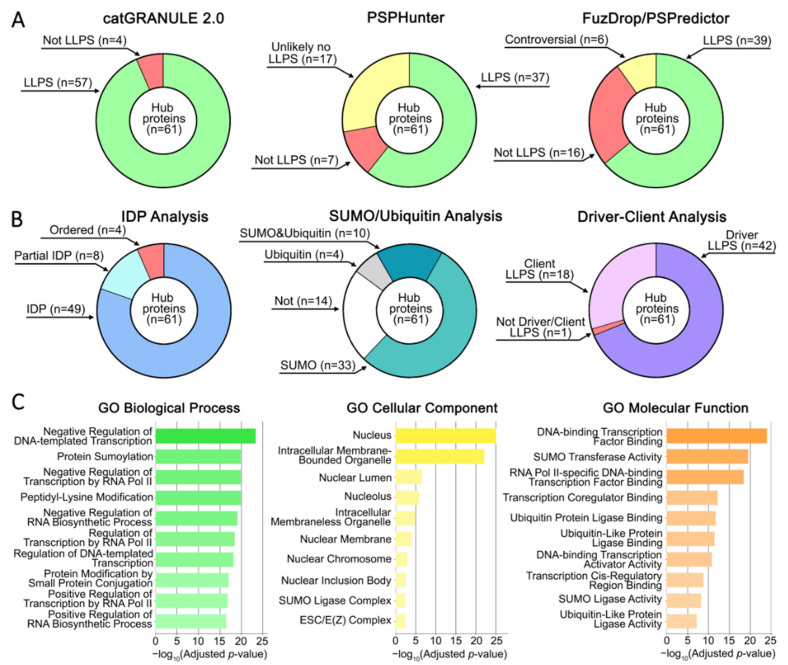
Comprehensive analysis of identified hub proteins. (**A**) Results of liquid–liquid phase separation (LLPS) propensity analysis using the following computational approaches: catGRANULE 2.0, PSPHunter, and FuzDrop/PSPredictor. (**B**) Results of intrinsically disordered protein (IDP) scoring (VSL2Score), SUMO/ubiquitin post-translational modifications (as annotated in the UniProt database), and propensity to function as LLPS drivers or clients. (**C**) Gene Ontology (GO) enrichment analysis: Biological Process, Cellular Component, and Molecular Function. Results are displayed as horizontal bar charts representing the 10 terms with the lowest *p*-values. The complete results are provided in Supplementary Table S3.

An overview of the bioinformatic properties of the identified hub proteins is presented in Figure 7. Of the 61 proteins: 39 show propensity for liquid–liquid phase separation (LLPS), 60 are predicted clients or drivers of LLPS, 57 are fully or partially intrinsically disordered, 43 are predicted SUMOylation substrates, and 14 are predicted ubiquitination substrates. Importantly, all three independent LLPS-prediction approaches consistently indicated phase separation propensity in more than half of the hub proteins.

This is particularly relevant given that PML bodies are membraneless organelles whose multi-step biogenesis is thought to involve LLPS [14,40]. Taken together, these findings point to a potentially complex and multifaceted network of molecular functions mediated by USP7 and highlight PML-NBs as an important cellular context for understanding the mechanisms underlying USP7 activity.

### 2.4. Selected Hub Proteins and Their Known Roles in USP7 and PML-NB Biology

**Figure 8:**
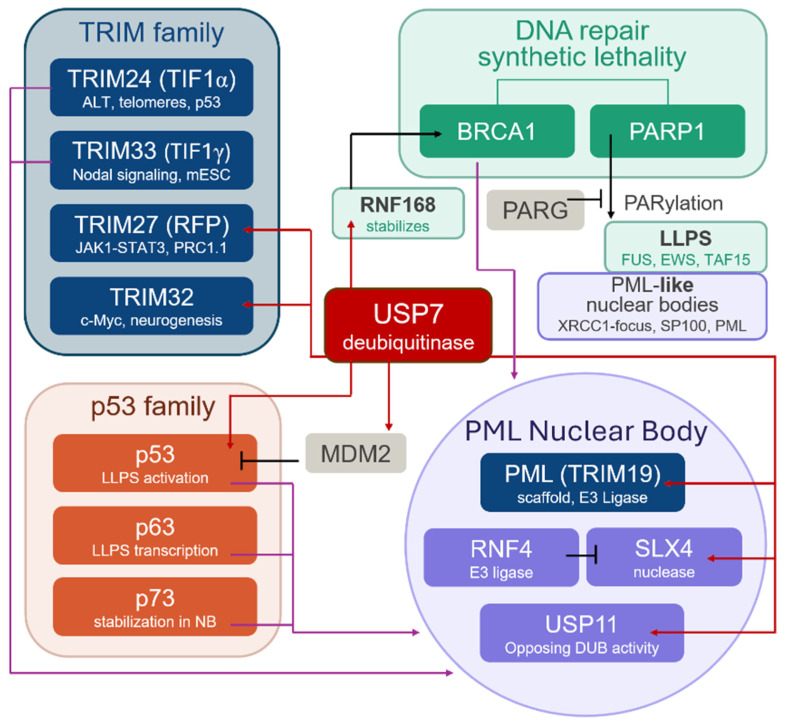
USP7 as a proposed regulatory hub at PML-NBs. Three functional groups are highlighted: the TRIM protein family (TRIM24, TRIM27, TRIM32, TRIM33, PML; blue), the p53 transcription factor family (p53, p63, p73; orange), and DNA repair/synthetic lethality components (BRCA1, PARP1). Red arrows show direct interactions with USP7; pink arrows indicate PML-NB localization; black arrows represent pathway activation; blunt-ended connectors indicate inhibitory/antagonistic relationships.

Among the identified hub proteins, a subset formed functionally coherent groups that are well represented in the literature and collectively suggest a broader biological context for USP7 activity at PML-NBs. USP7 is a deubiquitinase with a wide range of substrates and interaction partners. Growing evidence suggests that USP7 functions not only as an autonomous regulatory enzyme, but also as an integral component of diverse molecular complexes. Three such groups were selected for closer examination: DNA repair and synthetic lethality—PARP1 and BRCA1; the p53 transcription factor family—p53, p63, and p73; and the TRIM protein family—TRIM19 (PML), TRIM27, TRIM33, and TRIM24 (Figure 8). The known properties of these proteins and their interactions with USP7, PML and PML-NBs are discussed below.

#### 2.4.1. PARP1, BRCA1

It is known that in response to DNA double-strand breaks (DSBs), repair proteins accumulate at damaged sites, forming membraneless condensates or “foci”. The formation of these foci and their timely disassembly within an appropriate temporal window are essential for genome integrity; however, the mechanisms underlying their assembly, maintenance, and dissolution remain unclear [41]. PARP1 and BRCA1 are key proteins in DNA repair mechanisms, and their interaction underlies the concept of “synthetic lethality”—a phenomenon in which the individual loss of either gene is compatible with cell viability, while simultaneous loss of both genes results in cell death [42,43].

It has recently been demonstrated that PARP1 does not form liquid–liquid phase separation (LLPS) condensates autonomously. Rather, PAR chains initiate LLPS for several intrinsically disordered proteins (FUS, EWS, and TAF15) at DNA break sites—a process tightly regulated upon dePARylation by PARG [41]. Following USP7 knockdown, elevated levels of cleaved PARP1 are observed, although this likely occurs indirectly through the apoptotic cascade (via the p53/MDM2 axis and/or caspase-7) rather than through direct interaction [44]. Although direct co-localization of PARP1 and PML has not yet been demonstrated, PARP1 has been shown to localize within XRCC1-containing foci, which have been identified as “PML-like subnuclear bodies” containing SP100 (a marker of PML bodies) and PML protein itself [45].

LLPS also has been shown for RAP80 protein to enhance the recruitment of BRCA1 to DNA double-strand breaks. RAP80 uses an intrinsically disordered region (IDR) to form such condensates, which are required for proper BRCA1 foci formation at damage sites [46]. Notably, BRCA1 has been shown to co-localize with both enlarged PML bodies and canonical PML-NBs [47]. USP7 also deubiquitinates and stabilizes RING finger protein 168 (RNF168), thereby rescuing the formation of ionizing radiation-induced foci (IRIF) and UV radiation-induced foci (UVRIF) of polyubiquitinated histone H2A and BRCA1, and promoting ubiquitin-dependent DNA damage signaling [8].

#### 2.4.2. P53, P63, P73

A central regulator of all TP53 family members is the promyelocytic leukemia protein (PML), which recruits TP53, TP63, and TP73 to nuclear subdomains—PML-NBs [48]. It has been observed that co-localization of p73 and PML within PML-NBs is required for p73 stabilization [49]. Furthermore, PML coordinates a number of critical regulators of TP63/TP73 and establishes cross-talk among them to sustain their transcriptional activity in response to stress. The p53 protein is capable of forming liquid-like condensates through LLPS, which may serve as a mechanism for regulating its activity [50]. It has been proposed that compartmentalization of p53 into droplets under normal cellular conditions suppresses its function as a transcriptional regulator, whereas p53 activation under stress conditions releases p53 from these droplets and promotes its transcriptional activity [50]. Notably, both P63 and p73 also undergo liquid–liquid phase separation (LLPS) [51].

#### 2.4.3. PML (TRIM19)

The PML protein (TRIM19) is the main component of PML-NBs—dynamic, membraneless nuclear organelles involved in the regulation of apoptosis, antiviral response, DNA repair, and transcription. Co-immunoprecipitation studies have demonstrated that USP7 physically interacts with PML, exhibiting a pronounced preference for the PML-IV isoform [15]. Since PML-IV differs from other isoforms exclusively in its intrinsically disordered C-terminal region (IDR) [14], this region is presumed to mediate the interaction with USP7, although strong interaction via a bridging partner cannot be excluded. As noted above, USP7 forms discrete nuclear foci and can co-localize with PML-NBs. The functional consequences of the USP7-PML interaction are complex and still unclear.

On one hand, USP7 knockdown leads to an increased number of PML-NBs, accumulation of PML protein, and suppression of its polyubiquitination, which indicate a direct enzymatic role for USP7. On the other hand, overexpression of USP7 in p53-null cells causes PML-NBs dissociation independently of the enzyme’s catalytic activity [15]. Thus, USP7 may regulate PML-NBs through two independent mechanisms: a catalytic mechanism (via ubiquitin chain modulation) and a non-catalytic mechanism (structural reorganization of PML-NBs through recruitment of additional binding partners).

It is also worth noting that PML regulation is not exclusive to USP7—USP11 is likewise capable of modulating PML. USP7 and USP11 show opposing regulatory effects on PML and physically interact with each other, potentially functioning as a complex to coordinate control over shared partners [52].

Of particular relevance are recent data [38] describing a role for PML-NBs in maintaining the stability of the scaffold protein SLX4—a regulator of the nucleases SLX1, MUS81, and XPF involved in DNA repair. Because uncontrolled nuclease activity poses a threat to genome integrity, the abundance and localization of SLX4 must be strictly regulated. It has been shown that the E3 ligase RNF4 mediates ubiquitin-dependent proteasomal degradation of excess SLX4 under normal conditions, while USP7, localized within PML-NBs, counteracts this process by stabilizing SLX4 and maintaining the cellular pool required for rapid DNA damage responses. The coordinated activity of RNF4 and USP7 within PML bodies thus constitutes a mechanism of spatial compartmentalization of nuclease activity—an interesting example of how PML bodies function as regulatory hubs and how USP7 serves as a key participant in maintaining ubiquitination homeostasis within these structures.

#### 2.4.4. TRIM27, TRIM33, TRIM24, and TRIM32

The E3 ubiquitin ligase TRIM27 (Ret Finger Protein, RFP) forms a stable complex with USP7 [53], the functional significance of which has been demonstrated in several biological contexts. In hepatocellular carcinoma cells, the TRIM27-USP7 complex drives activation of the JAK1-STAT3 pathway: USP7 stabilizes TRIM27 through deubiquitination, while the resulting ternary JAK1-TRIM27-USP7 complex sustains oncogenic signaling, thereby promoting epithelial-to-mesenchymal transition and the acquisition of stem-like properties by tumor cells [54]. In myeloid leukemia, USP7 and TRIM27 are structural components of the non-canonical PRC1.1 Polycomb complex, where USP7 neutralizes the E3 ligase activity of TRIM27, preserving complex integrity and transcriptional function. Additionally, both TRIM27 and USP7 are required for incorporation into PRC1.1 [55].

The E3 ubiquitin-protein ligase TRIM33 (Transcription Intermediary Factor 1-gamma, TIF1γ) co-localizes with PML-NBs in mouse embryonic stem cells (mESCs), where it mediates Nodal signaling-directed transcription of Lefty1/2 [56]. Notably, TRIM33 condensate formation in mESCs is strictly dependent on PML protein expression and PML-NBs assembly. TurboID proximity-labeling data confirms strong enrichment of TRIM33 specifically within mESC-specific PML-NBs, pointing to the cell-context-dependent composition of PML bodies. This work further confirms that PML-NBs recruit distinct sets of client proteins depending on cell type and cellular state, rather than representing static structures with a fixed molecular composition.

TRIM24 (Transcription Intermediary Factor 1-alpha, TIF1α) functions both as a transcriptional co-activator that reads histone H3 modifications and as an E3 ubiquitin ligase involved in TP53 degradation [57]. TRIM24 localizes with PML-NBs together with PIAS4, TRIM33, and UBC9, forming a functional cluster of SUMO/ubiquitin regulatory factors [58]. A role for TRIM24 has also been described in coordinating the alternative lengthening of telomeres (ALT) mechanism through acetyl-dependent recruitment to telomeres and organization of ALT-associated PML bodies (APBs)—a specialized subpopulation of altered PML-NBs [59]. Notably, tethering of TRIM24 to telomeres proved sufficient to stimulate de novo telomeric DNA synthesis in a SUMO-dependent, but p300/CBP- and PML-independent, manner.

TRIM32 is an E3 ligase for c-Myc and a regulator of neuronal differentiation. USP7 has been shown to physically interact with TRIM32 and to antagonize its activity by deubiquitinating and stabilizing c-Myc, thereby maintaining the stem cell state of neural stem cells. Furthermore, TRIM32 is upregulated during adult neurogenesis [60]. Although direct association of TRIM32 with PML-NBs has not been demonstrated, evidence indicates that TRIM32 can influence the subnuclear localization of partner proteins (notably Glis2) adjacent to PML bodies [61]. Thus, the TRIM32-USP7 axis, analogously to the TRIM27-USP7 pair, balances ubiquitination and deubiquitination of key transcription factors.

Taken together, these findings support a unifying concept: USP7 may function as a multilevel regulator within the regulatory landscape of TRIM family proteins, operating predominantly within or in close functional association with PML-NBs. The interaction of USP7 with TRIM proteins (TRIM19/PML, TRIM27, TRIM24, TRIM33, and TRIM32) is mediated through diverse molecular mechanisms—ranging from direct substrate deubiquitination to structural reorganization of multiprotein complexes and protein–protein interactions. The cell context-specificity of these interactions determines the resulting biological outcome: maintenance of stemness, oncogenic progression, DNA damage response, or transcriptional regulation. Based on the present analysis, PML-NBs may act not only as passive sites of protein localization, but as active spatial regulators that modulate the availability and activity of components within TRIM-USP7 signaling networks in a cell type- and functional state-dependent manner.

## 3. Materials and Methods

### 3.1. Study Design and Databases

The interactome lists for USP7, PML, core PML-NB-associated proteins, and Cajal body-associated proteins were compiled as follows:

i. Interactome lists for USP7, PML, UBC9, CREBBP, MDM2, PIAS1, P53, SUMO1, DAXX, and SLX4 were retrieved from the open-access protein–protein interaction database BioGRID v.5.0 (https://thebiogrid.org/, accessed on 25 February 2026) [62].
ii. A literature-derived list of 205 proteins reported to localize to PML nuclear bodies by fluorescence or electron microscopy was used [39].
iii. The Cajal body proteome was retrieved from the UniProt database (https://www.uniprot.org/locations/SL-0031, Filter: Homo sapiens (Human); accessed on 27 February 2026) [63].

The CellAge and GenAge databases were used for cellular senescence and aging gene data analysis, respectively (https://genomics.senescence.info/cells/; https://genomics.senescence.info/genes/, accessed on 27 February 2026) [64,65].

**Figure 9:**
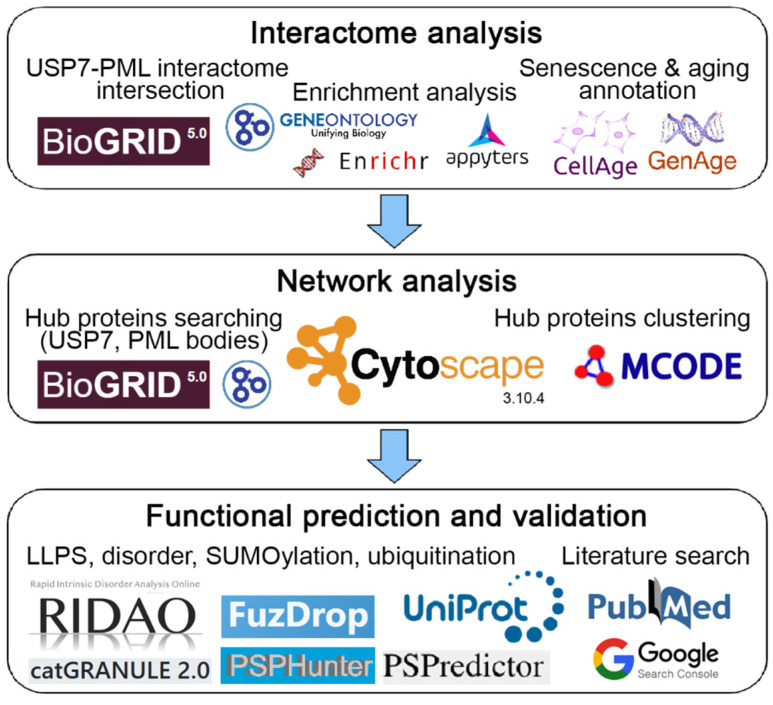
Scheme illustrating the design of this study.

Figure 9 schematically represents the design of our study. Summary tables for all analyses performed are provided in the Supplementary Materials.

### 3.2. Enrichment Analysis

Reactome Pathway, DisGeNET [66], and Gene Ontology (GO) analyses were performed using Enrichr (https://maayanlab.cloud/Enrichr/, accessed 25 February 2026), and the 10 terms with the lowest *p*-values were selected [67,68]. The adjusted *p*-values were calculated automatically by the platform. The following metrics were included in the Supplementary Materials: *p*-value, Adjusted *p*-value (Benjamini–Hochberg correction), Odds Ratio, Combined Score (log(*p*-value) × z-score), and the list of overlapping genes. To retrieve the enrichment analysis results and download the full report, the Appyter “Enrichment Analysis Visualizer” (https://appyters.maayanlab.cloud/Enrichment_Analysis_Visualizer/, accessed on 25 February 2026) was used [69].

### 3.3. LLPS Predisposition Analysis

Three approaches were used to assess LLPS predisposition:

i. catGRANULE 2.0 (https://tools.tartaglialab.com/catgranule2, accessed on 25 February 2026)—an accurate predictor of LLPS-prone proteins at single amino acid resolution. A threshold of >0.5 was applied to discriminate LLPS from non-LLPS proteins, as defined in the original publication [70].
ii. PSPHunter (http://psphunter.stemcellding.org/, accessed on 25 February 2026)—a machine learning-based method designed to predict phase-separating proteins and their corresponding driving residues. The following score thresholds were used for LLPS predisposition classification, in accordance with the original documentation and the associated publication [71]: score > 0.61—likely to undergo phase separation; score 0.36–0.61—unlikely to undergo phase separation; score < 0.36—non-LLPS.
iii. A combined approach integrating two predictors: FuzDrop [72] (https://fuzdrop.bio.unipd.it/, accessed on 25 February 2026) and PSPredictor [73] (http://www.pkumdl.cn:8000/PSPredictor/, accessed on 25 February 2026). Proteins exhibiting a PSPredictor score > 0.5 and a FuzDrop score > 0.6 were classified as having LLPS propensity. In cases where FuzDrop and PSPredictor analyses yielded conflicting results, the respective proteins were assigned to the “controversial LLPS” group [39].

FuzDrop enables prediction of a protein’s propensity for both spontaneous and interaction-induced LLPS. Proteins capable of undergoing phase separation spontaneously are classified as droplet-driving proteins (drivers), whereas those requiring additional intermolecular interactions are termed clients. Proteins with a pLLPS ≥ 0.60 are predicted to drive LLPS, while those containing droplet-promoting regions—defined as consecutive residues with pDP ≥ 0.60—are predicted to function as droplet clients [39].

### 3.4. Intrinsic Disorder Prediction

The propensity of the studied proteins for intrinsic disorder was performed using the RIDAO platform (https://ridao.app/, accessed on 25 Febrary 2026), which employs a set of algorithmic predictors: PONDR^®^ FIT, PONDR^®^ VLXT, PONDR^®^ VLS2, PONDR^®^ VL3, IUPred-Long, and IUPred-Short [39,74]. The percentage of predicted intrinsically disordered residues (PPIDR), derived from the PONDR^®^ VSL2 algorithm and defined as the fraction of residues with a disorder score > 0.5, was used as the primary indicator. PPIDR values below 10% are characteristic of highly ordered proteins, values in the range of 10–30% indicate moderately disordered proteins, and values above 30% are associated with highly disordered proteins [75].

### 3.5. Protein SUMOylation, Ubiquitination and Characterization

Protein SUMOylation and ubiquitination analysis was performed by querying the UniProt database (https://www.uniprot.org/, UniProt KB accessed on 27 February 2026) [63] and identifying mentions of SUMO or ubiquitination within the PTM/Processing section of the corresponding UniProt entry. Hydrophobicity and charge at pH 7.0 were assessed using Biopython 1.87 (Bio.SeqUtils package, Bio.SeqUtils.ProtParam module), using the charge_at_pH and GRAVY functions [76]. The charge_at_pH function computes the charge of a protein at a specified pH value, while the GRAVY (Grand Average of Hydropathicity) index was calculated to estimate hydrophobicity according to Kyte and Doolittle’s work [77].

### 3.6. Protein–Protein Interaction Network and Clustering

Protein–protein interaction (PPI) network construction and visualization, including cluster representation, were performed using Cytoscape v.3.10.4 [78]. Proteins interacting with ≥4 core PML-NB components were classified as hubs. This threshold was selected based on the following reasoning: interactions with 1–2 proteins were considered insufficient to reliably indicate PML-NB association; a threshold of 3 was also considered insufficient, as the majority of such associations exhibited low BioGRID evidence scores and were disproportionately attributable to TP53—a protein with an anomalously large interactome (>2500 partners). A threshold of ≥4 is consistent with the lower bound reported in proteomic hub-definition studies [79,80] and is statistically unlikely to arise by chance within a restricted organelle-specific dataset.

Network clustering was carried out using the MCODE algorithm [81] with the following parameters: degree cutoff = 2, node score cutoff = 0.2, k-core = 2, max depth = 100, haircut = false, fluff = false. Functional enrichment analysis of the identified clusters was performed using the enrichment analysis tool of the STRING database [82], which includes the Gene Ontology, KEGG, and Reactome pathways categories.

### 3.7. Plasmids

Plasmids carrying genes encoding fusion proteins of seven PML isoforms (I–VII) with green fluorescent protein (EGFP), based on the pEGFP-C1/3 vectors, were obtained as described in [83] and were used for transient transfection. The human USP7, UBC9, and MDM2 genes were isolated using cDNA obtained by reverse transcription of mRNA isolated from the blood of a healthy donor. The UBC9 and MDM2 genes were amplified using corresponding primers and then cloned into the pTag-GFP2-C vector (Evrogen, Moscow, Russia) via restriction-ligation. The resulting constructs encoding the UBC9-EGFP and MDM2-EGFP fusion proteins were used for transient transfection.

The fluorescent USP7-RFP construct used for lentiviral transfection was based on the pUltraHot vector (a gift from Malcolm Moore, Addgene plasmid #24130) in which the mCherry gene was replaced with the puromycin resistance gene [84]. The red fluorescent protein gene (RFP) was amplified from pTag-RFP-C (Evrogen, Moscow, Russia). The USP7 and RFP genes were amplified using corresponding primers and then cloned into the pUltraHot vector via restriction-ligation. Plasmids for the lentiviral vector system, psPAX2 and pMD2.G, were a gift from Didier Trono (Addgene #12260 and #12259).

All plasmids were prepared by transforming the corresponding constructs into *Escherichia coli* DH5α cells (for transient transfection constructs) or Stbl3 cells (for the lentiviral system constructs) by electroporation. Several clones were selected for sequencing. Plasmid isolation was performed using the Plasmid Miniprep Kit (Evrogen, Moscow, Russia). All constructs were sequenced using the BigDye Terminator v3.1 Cycle Sequencing Kit (Thermo Fisher Scientific, Waltham, MA, USA), and the samples were analyzed using the ABI PRISM 3500 genetic analyzer (Applied Biosystems, Foster City, CA, USA).

### 3.8. Cell Culture and Confocal Microscopy

HeLa, U2OS and HEK293T cell lines were kindly provided by the cell culture collection of the Institute of Cytology RAS. The cell lines were cultured in DMEM medium (Biolot, Saint-Petersburg, Russia) supplemented with 10% FBS (Cytiva, Marlborough, MA, USA), L-glutamine (Biolot, Saint-Petersburg, Russia), and penicillin–streptomycin (Biolot, Saint-Petersburg, Russia). The cells were maintained at 37 °C and 5% CO_2_ in a humidified incubator.

Stable HeLa and U2OS cell lines expressing the USP7-RFP fusion protein were created using a lentiviral vector system. HEK293T cells at 70% confluency were co-transfected with the pUltraHot-USP7-RFP, psPAX2, and pMD2.G plasmids using PEI MAX (Polysciences, Warrington, PA, USA) as a transfection reagent. After 48 h of incubation at 37 °C, 5% CO_2_, the supernatant was collected, centrifuged at 300× *g* for 5 min, filtered through a 0.45 µm PES filter unit, and added to the cells. After 24 h, the medium was changed, and selective antibiotic puromycin (5 μg/mL, InvivoGen, San Diego, CA, USA) was added for 7 days. After selection, the stable cell lines were confirmed using a fluorescent microscope.

Live cell imaging was performed by plating cells 35 mm glass Ibidi dishes on pre-treated with poly-L-lysine (Sigma-Aldrich, St. Louis, MO, USA). After 24 h, cells were transfected with plasmids using GeneJect 40 (Molecta, Moscow, Russia). The cells were visualized by irradiating them with a laser at wavelengths of 488 nm and 561 nm to excite EGFP or RFP fluorescence, respectively. The resulting fluorescence was recorded using an Olympus FV3000 confocal microscope (60× oil immersion objective, NA 1.42). Images were corrected for background signal and high-frequency noise using ImageJ software (Version 1.53c).

## 4. Conclusions

USP7 is a deubiquitinating enzyme with a large number of substrates and interaction partners, making it a promising target for therapeutic development. Growing evidence suggests that USP7 functions not only as an autonomous regulatory enzyme, but also as a component of diverse molecular complexes. However, the molecular mechanisms underlying USP7 colocalization with PML-NBs and the functional significance of this association remain uncharacterized. In this study, comparative analysis of the USP7 and PML interactomes revealed 166 shared protein partners, extensive molecular coupling between USP7 and PML-NBs at the level of protein–protein interactions. Functional enrichment analysis demonstrated that both proteins operate within a common molecular context related to transcriptional regulation, chromatin remodeling, and transcriptional responses to DNA damage; according to the CellAge and GenAge databases, these processes are linked to mechanisms of cellular senescence and organismal aging.

Localization analysis demonstrated that USP7 colocalizes with nuclear PML isoforms (PML-I through PML-VI) within PML-NBs, but not with the cytoplasm-localized PML-VII. USP7 nuclear foci were also found to colocalize with UBC9 and MDM2, consistent with USP7’s roles as a SUMO deubiquitinase and MDM2 stabilizer, respectively. Notably, co-expression with PML isoforms produced larger nuclear USP7 foci compared to UBC9 or MDM2, implying distinct modes of USP7 incorporation into PML-NBs depending on the interacting partner.

Focused analysis of the overlap between the USP7 interactome and the core PML-NBs-associated proteome identified 61 hub proteins forming a dense network with small-world topology: the vast majority of these proteins are prone to liquid–liquid phase separation, are fully or partially intrinsically disordered, and serve as substrates for SUMOylation and ubiquitination. The presence of several TRIM family members—TRIM19 (PML), TRIM27, TRIM33, and TRIM24—together with PARP1, BRCA1, and p53 family proteins p53, p63, and p73, suggests that USP7 may function as a multilevel regulator within the TRIM protein ecosystem, predominantly in the context of PML-NBs or in close functional association with them. These findings are based on bioinformatic analyses and therefore represent predicted rather than experimentally validated associations. Nevertheless, their internal consistency and specificity define a clear framework for hypothesis-driven investigation across several interconnected directions.

Mechanistic questions concern USP7 recruitment itself: co-immunoprecipitation, proximity ligation assays, and domain mutagenesis would map the interaction interface, while live-cell imaging would resolve colocalization dynamics. Given the prevalence of LLPS-prone, disordered hub proteins, USP7 may regulate the condensation and dissolution of PML-NBs. Because hub proteins are dual substrates for SUMO and ubiquitin, USP7 likely operates at the crossroads of that crosstalk; ubiquitinome and SUMOylome profiling under USP7 loss, combined with competitive interaction studies with RNF4, would clarify how USP7 balances these opposing marks. Systematic mapping of USP7 interactions across the identified TRIM proteins, integrated with USP7 ChIP-seq and TRIM-specific binding profiles, would further illuminate how deubiquitination controls E3 ligase activity and genomic targeting.

Stress-response questions follow from the overlap with DNA damage components. Potentially, the USP7-PML axis may influence repair pathway choice and apoptotic decisions after genotoxic stress; CRISPR-based screens combined with high-resolution imaging could identify which factors mediate stress-dependent USP7 recruitment and how this affects p63/p73 stability. The connection of the 166 shared proteins to senescence databases warrants testing whether USP7-PML interactions are remodeled during oncogene-induced or stress-induced senescence.

Research priorities include evaluating the effects of clinical USP7 inhibitors on PML-NB integrity, screening for molecules that selectively disrupt the USP7-PML interface and exploring synergism between USP7 inhibition and PML-degrading agents such as arsenic trioxide or interferons. Among all directions, the intersection of LLPS biology, deubiquitination, and PML-NB organization stands out as particularly compelling: it bridges two rapidly advancing fields in a system where experimental data are extremely limited.

Taken together, these findings suggest that USP7 may extend its regulatory reach through integration into the PML-NB interactome, and point toward a coherent experimental framework—from condensate biology and PTM crosstalk to therapeutic intervention—for exploring its context-dependent functions.

## Figures and Tables

**Table 1:** USP7 subcellular localization and functional partnerships.

Localization	Partner(s)	Functional Effect	References
PML nuclear bodies (PML-NBs)	PML PML-IV PML-I	USP7 destabilizes PML-NBs through its N- and C-terminal domains via interactions with PML proteins (preferentially PML-IV); USP7 silencing led to increased levels of PML isoforms I, II, and IV.	[15]
	DAXX MDM2 P53	DAXX and MDM2 co-localize within PML-NBs and physically interact with one another. DAXX forms a ternary complex with MDM2 and USP7, thereby participating in the regulation of the MDM2/p53 pathway.	[6,16,17,18,27]
Replication forks	MCM4 SUMO2	USP7 is enriched at replication forks as a replisome-associated deubiquitinase, where it interacts with MCM4, but does not directly interact with PCNA. USP7 deubiquitinates SUMO2 and SUMOylated proteins, thereby maintaining a SUMO-enriched, ubiquitin-depleted environment at active replication forks.	[19]
Telomeric ends, ALT-associated PML bodies (APBs)	TPP1 TSPYL5	USP7 localizes to telomeres in both telomerase-positive and ALT-positive cells, where it interacts with shelterin components, primarily via deubiquitination of TPP1. In ALT-positive cells, USP7 activity depends on the presence of PML and is regulated by TSPYL5, which competitively inhibits USP7 substrate binding.	[9,21]
Cajal bodies *	WDR79	USP7 interacts with WDR79, a key component of Cajal bodies, resulting in reduced ubiquitination of MDM2 and p53, thereby stabilizing these proteins.	[22,28]
Cytoplasm	TRIM27 Raf-1, TFEB TRAF6, IKKγ	The functional significance of cytoplasmic USP7 pools is not yet fully understood. In the cytoplasm, USP7 has been shown to form a complex with TRIM27 and to interact with Raf-1, TFEB, TRAF6, and IKKγ.	[29,30,31,32]

* No information about USP7 localization within Cajal bodies has been reported.

## Data Availability

Data are contained within the article or Supplementary Materials.

## Supplementary Materials

The following supporting information can be downloaded at: https://www.mdpi.com/article/10.3390/ijms27094106/s1.

## Abbreviations

The following abbreviations are used in this manuscript:

USP7	Ubiquitin-specific protease 7
HAUSP	herpesvirus-associated ubiquitin-specific protease, alternative USP7 protein name
PML	promyelocytic leukemia protein
PML-NB	PML nuclear body
UPS	ubiquitin-proteasome system
DUB	deubiquitinating enzyme
USP	ubiquitin-specific protease
SUMO	Small Ubiquitin-like Modifier
PTEN	phosphatase and tensin homolog deleted on chromosome 10 protein
NF-κB	Nuclear factor kappa-light-chain-enhancer of activated B cells
FOXP3	Forkhead box protein P3
DAXX	Death domain-associated protein 6
PCNA	Proliferating cell nuclear antigen
ALT	Alternative Lengthening of Telomeres
APBs	ALT-associated PML bodies
POT1	Protection of telomeres protein 1
WDR79	WD repeat-containing protein 79
GO	gene ontology
RNA	Ribonucleic acid
IDP	Intrinsically Disordered Proteins
DPR	disorder-promoting residues
LLPS	liquid–liquid phase separation
DSBs	DNA double-strand breaks
