# Supplementary figures and images for: USP7 at PML Nuclear Bodies: A Protein Interaction Network Perspective
Source: Int J Mol Sci. 2026 May 4;27(9):4106. doi: 10.3390/ijms27094106 (PMC13164317; doi:10.3390/ijms27094106)

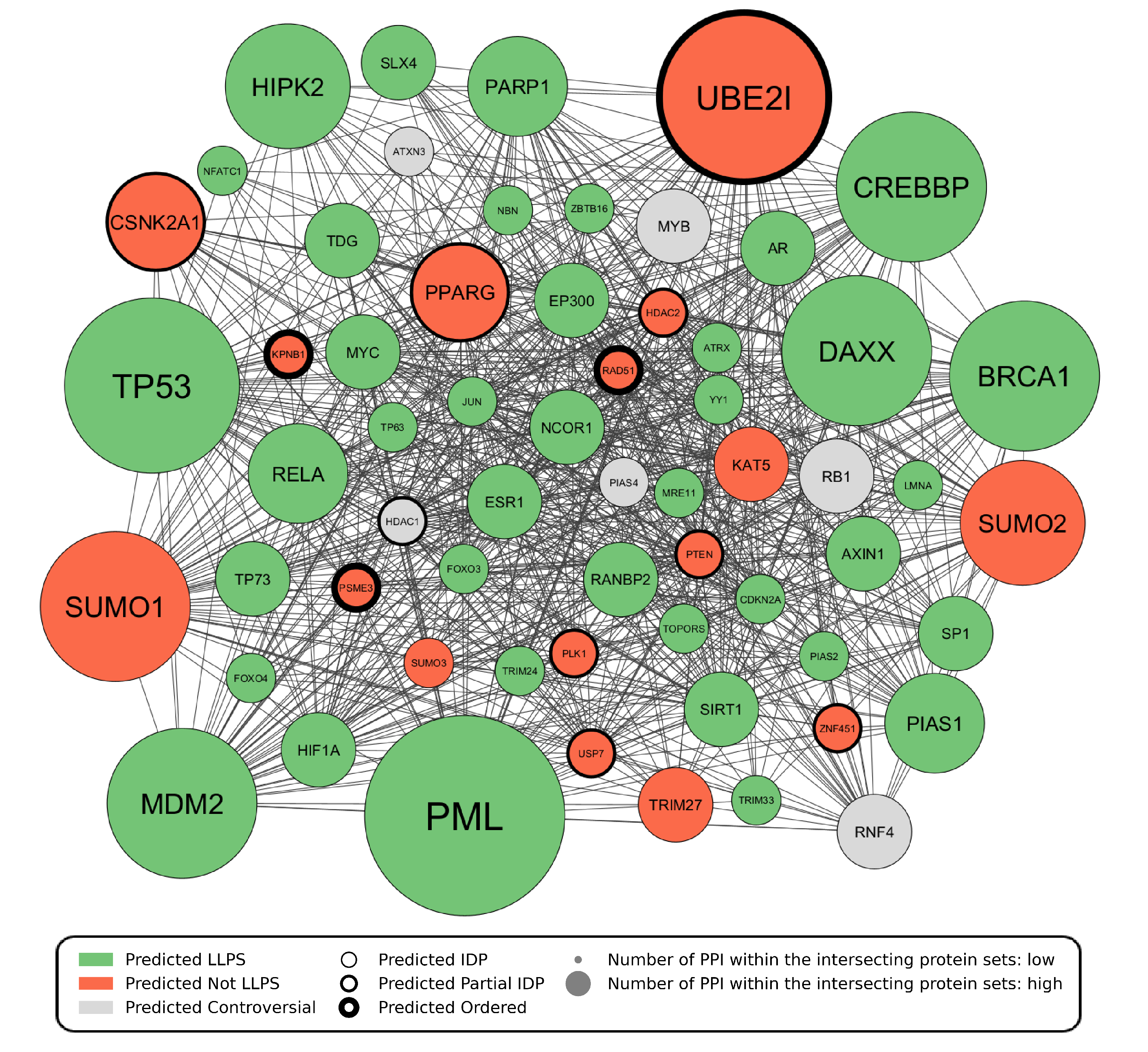

Supplement: Supplementary file 1 [file ijms-27-04106-s001.zip › FigureS1.tif]
